# Effectiveness of papain gel in venous ulcer treatment: randomized clinical trial^1^

**DOI:** 10.1590/0104-1169.0381.2576

**Published:** 2015-07-03

**Authors:** Ana Luiza Soares Rodrigues, Beatriz Guitton Renaud Baptista de Oliveira, Débora Omena Futuro, Silvia Regina Secoli

**Affiliations:** 2 MSc, RN, Hospital Federal da Lagoa, Rio de Janeiro, RJ, Brazil; 3 PhD, Full Professor, Escola de Enfermagem, Universidade Federal Fluminense, Niterói, RJ, Brazil; 4 PhD, Associate Professor, Faculdade de Farmácia, Universidade Federal Fluminense, Niterói, RJ, Brazil; 5 PhD, Associate Professor, Escola de Enfermagem, Universidade de São Paulo, São Paulo, SP, Brazil

**Keywords:** Leg Ulcer, Papain, Carboxymethylcellulose Sodium, Wound Healing, Clinical Trial, Nursing

## Abstract

**OBJECTIVE:**

to assess the effectiveness of 2% papain gel compared to 2% carboxymethyl cellulose in the treatment of chronic venous ulcer patients.

**METHOD:**

randomized controlled clinical trial with 12-week follow-up. The sample consisted of 18 volunteers and 28 venous ulcers. In the trial group, 2% papain gel was used and, in the control group, 2% carboxymethyl cellulose gel.

**RESULTS:**

the trial group showed a significant reduction in the lesion area, especially between the fifth and twelfth week of treatment, with two healed ulcers and a considerable increase in the amount of epithelial tissue in the wound bed.

**CONCLUSION:**

2% papain gel demonstrated greater effectiveness in the reduction of the lesion area, but was similar to 2% carboxymethyl cellulose gel regarding the reduction in the amount of exudate and devitalized tissue. Multicenter research is suggested to evidence the effectiveness of 2% papain gel in the healing of venous ulcers. UTN number: U1111-1157-2998

## Introduction

Chronic ulcer treatment, mainly in case of venous ulcers, represents a considerable strain for health services^1^^-^ 2. Specialized human resources and adjuvant therapies are needed, which are frequently adopted for lengthy treatment. In addition, this type of lesion often affects the patient's functional capacity, causing multidimensional consequences - social, psychological, financial^3^.

Venous ulcers represent the most advanced stage of chronic venous insufficiency. The global prevalence rate ranges between 0.5 and 2%, and surpasses 4% in individuals over 65 years of age. Incidence rates vary between two and five new cases per thousand people per year^4^^-^ 5.

Studies developed in recent years have contributed to an important increase in the availability of new wound healing products^6^^-^ 9. Papain dressings have been studied in different formulations and concentrations as an option in venous ulcer treatment^10^.

In a systematic review aimed at analyzing evidence on the use of papain in the wound healing process, the results appointed the lack of a standard form and presentation to use the product, besides the predominance of low-quality research according to the international assessment scales, indicating the need to develop research with stricter methods for the sake of a more precise assessment of the effectiveness of papain in the tissue repair process^11^.

The study objective was to assess the effectiveness of 2% papain gel compared to 2% carboxymethyl cellulose in the treatment of chronic venous ulcer patients.

## Method

A Randomized Controlled Clinical Trial (RCCT) was developed at a specialized outpatient clinic in wound treatment of a teaching hospital in the State of Rio de Janeiro, Brazil.

The consecutive sample consisted of all male and female individuals who complied with the inclusion criteria, which were: age ≥18 years; presence of one or more venous leg ulcers with a minimum length of six weeks; indication to use 2% papain gel and 2% carboxymethyl cellulose gel; demonstrate understanding of the health team's orientations to guarantee the continuity of the treatment at home or having a legal responsible to do so; availability to visit the clinic once per week; being regularly registered at the institution where the research was undertaken.

Patients were excluded in the following cases: infected ulcer, associated with erysipelas, cellulitis or lymphangitis, ulcer with the presence of devitalized tissue covering the entire wound bed; with circular lower-limb lesions, with non-palpable distal pulse; without appropriate conditions to store the products at home; patients with a background history of alcoholism or psychiatric diseases, individuals allergic to any of the products used in the research and/or to latex; patients with liver and/or kidney problems.

The criteria for discontinuity were: presence of wound infection during follow-up, use of another product on the wound than that proposed in the research, occurrence of severe adverse events and not participating in weekly outpatient appointments.

The patients were recruited between April and November 2013 and monitored between April 2013 and January 2014.

The initial research sample consisted of 21 randomized volunteers allocated to two groups, 11 in the Trial Group and 10 in the Control Group. Three participants were lost due to absence from the weekly outpatient consultations, resulting in a sample of 18 patients, 10 in the Trial Group and 8 in the Control Group.

The patients who agreed to participate in the study and signed the Informed Consent Form were randomized and allocated to two groups: Trial Group (TG) and Control Group (CG). In the Trial Group, the ulcer dressing was applied using 2% papain gel and, in the Control Group, the product used was 2% carboxymethyl cellulose gel. For the sake of randomization, a research collaborator used a table with random figures. The study participants were only informed about which group the patient would be allocated to at the moment of each volunteer's first consultation.

In those situations when the patient had more than one lesion, all injuries were treated with the same product, according to the patient's allocation in the study.

The 2% papain gel was formulated using papain, EDTA, propylene glycol, carbopol 940, preservatives and purified water. The formula of the 2% carboxymethyl cellulose gel contained carboxymethyl cellulose, propylene glycol, preservatives and purified water. The products used in the research were developed at the university pharmacy.

The primary research outcome was the reduction in the lesion area, assessed by means of manual planimetrics to calculate the area in square centimeters. Digital photographic records were used as a complementary technique to assess the injury. The secondary outcomes were the reduction of the devitalized tissue in the wound bed and the reduction of the amount of exudate.

To assess the wounds, the Assessment Protocol for Clients with Tissue Injuries was used, an instrument used at the outpatient clinic which resulted from a research^12^. According to the protocol, the type of tissue was classified as granulation, epithelialization, slough and coagulation necrosis, and the amount of each tissue was registered as: absent; between 1 and 25%; between 26 and 50%; between 51 and 75% and between 76 and 100% of the total lesion area. To assess the effectiveness of the product to treat the lesions, the amount of each tissue was compared in the first and final week of follow-up.

As regards the exudate, the amount was assessed as: absent, when the wound bed was dry; little, when the wound bed was moist and involved drainage of at least 25% of the dressing; moderate, when the wound bed was saturated and the drainage involved between 25 and 75% of the dressing; and great, when the wound bed was covered with fluid and the drainage involved more than 75% of the dressing^13^^-^ 14.

The participants were assessed weekly and the planimetric records and photograph of the lesion were taken every two weeks. During the first visit, the data were collected on the patient's sociodemographic and health conditions. The researchers changed the dressing once per week at the wound clinic and the patients changed it daily at home. To apply the dressing at home, the patients received preliminary orientations, including information leaflets, on the dressing, storage and transportation of the products. Two nurse researchers performed the procedures for the two treatment groups.

The patients from the Trial and Control Groups received the material needed for the daily dressing change, the "dressing kit". This kit included a tube with 2% papain gel or 2% carboxymethyl cellulose gel, gauze, bandage, adhesive tape, 0.9% saline solution, soothing solution for the skin surrounding the lesion and a recipient to transport the product.

The length of the follow-up was 77 days/12 weeks, defined based on the mean number of days of international clinical experimental trials involving chronic ulcer patients^15-16^.

The blinding of the participants and researchers was compromised due to the product characteristics, such as refrigeration of the papain gel at 4ºC and maintenance of the carboxymethyl cellulose gel at room temperature. The evaluators of the result, who carried out the statistical analysis of the data, were blinded.

The chi-square test (c^2)^ for binomial variables was applied. The Friedman test was used for the association between an ordinal and a nominal variable for samples with more than two measures. In case of association between one ordinal and another dichotomous variable, the Mann-Whitney test was used for non-paired data and the Wilcoxon test for paired data. Mean posts were used to calculate the lesion area and multiple comparisons were made between the posts, considering different pairs of treatment weeks. The lesion areas were compared between the treatment weeks. Significance was set at 5%.

The research received approval from the Institutional Review Board at the Medical School, Hospital Universitário Antonio Pedro, Faculdade Fluminense de Medicina under number 196/98; CAAE 0154.0.258.000-08.

## Results

Eighteen patients participated in the research, including ten in the Trial Group and eight in the Control Group, totaling 28 ulcers that were treated during 12 weeks.

The Trial Group and the Control Group were homogeneous in terms of the sociodemographic and health characteristics, as presented in Table 1.

**Table 1.** Distribution of patients' sociodemographic and health variables according to the treatment group. Niterói, RJ, Brazil, 2014

Demographic and health variables	Category	Control Group		Trial Group		p-value*
		n=8	%	n=10	%	
Age	<60 years	4	50.0	4	40.0	1.000
	≥60 years	4	50.0	6	60.0	
Gender	Female	3	37.5	6	60.0	0.637
	Male	5	62.5	4	40.0	
Education	Primary education	6	75.0	6	60.0	0.638
	Secondary education	2	25.0	4	40.0	
Marital status	Married	4	50.0	6	60.0	1.000
	Not married	4	50.0	4	40.0	
Occupation	Employed	1	12.5	3	30.0	0.773
	Retired	6	75.0	6	60.0	
	Unemployed	1	12.5	1	10.0	
Individual monthly income^†^	Up to 1 minimum wage	7	87.5	4	40.0	0.087
	Between 1 and 2 minimum wages	0	0	4	40.0	
	Between 2 and 3 minimum wages	1	12.5	2	20.0	
Baseline diseases	CVI	3	37.5	4	40.0	0.342
	CVI + SAH	3	37.5	6	60.0	
	CVI + SAH + DM^‡^	2	25.0	0	0	
BMI^§^	Eutrophic	2	25.0	3	30.0	0.840
	Overweight	3	37.5	5	50.0	
	Obese	3	37.5	2	20.0	
Diet	Free	4	50.0	6	60.0	0.827
	Low-sodium	2	25.0	3	30.0	
	Low-sodium and low-sugar	2	25.0	1	10.0	

*Chi-square test, H0:PGC=PGE (equality of proportions between groups) vs. H1:PGC # PGE (difference of proportions between groups) †Federal minimum wage R$678.00, 2013, Brazil ‡CVI (chronic venous insufficiency); SAH (systemic arterial hypertension); DM (diabetes mellitus) §Body Mass Index (BMI), data regarding BMI classification from World Health Organization

The patients' age ranged between 45 and 85 years, with a mean age of 61.94 years and standard deviation ±12.5. In the 18 participants, 28 venous ulcers were identified, as there were patients with more than one injury, and five was the largest number of injuries in the same patient. Sixteen lesions were included in the Trial Group and 12 in the Control Group.

As regards the characteristics of the venous ulcers, the majority started more than ten years earlier (53.6%), 3.6% between 7 and 10 years, 32.1% had been open for between 4 and 6 years and 10.7% less than 3 years. The most affected location was the ankle region (53.6%), and the prevalent lower limb was the left (64.3%).

The lesion area of the patients in the Trial Group, when compared between the treatment weeks, showed a significant difference (p=0.006). The lesions in the Control Group showed no statistically significant difference in the area between the treatment weeks, with a p-value of 0.408.

To assess when the main variation took place among the treatment weeks in the Trial Group, multiple comparisons were made among the lesion areas, considering different pairs of treatment weeks (Table 2).

**Table 2.** Comparison of lesion areas in the Trial Group, considering different treatment weeks (n=16). Niterói, RJ, Brazil, 2014

Pairs of treatment weeks	p-value*	Adjusted p-value+
12^th^ week – 9^th^ week	0.741	1.000
12^th^ week – 7^th^ week	0.321	1.000
12^th^ week – 5^th^ week	0.002	0.032
12^th^ week – 3^rd^ week	0.016	0.240
12^th^ week – 1^st^ week	0.023	0.350
9^th^ week – 7^th^ week	0.508	1.000
9^th^ week – 5^th^ week	0.006	0.092
9^th^ week – 3^rd^ week	0.038	0.565
9^th^ week – 1^st^ week	0.053	0.791
7^th^ week – 5^th^ week	0.038	0.565
7^th^ week – 3^rd^ week	0.156	1.000
7^th^ week – 1^st^ week	0.202	1.000
5^th^ week – 3^rd^ week	0.508	1.000
5^th^ week – 1^st^ week	0.422	1.000
3^rd^ week – 1^st^ week	0.887	1.000

*Test of multiple comparisons (non-adjusted p-value). +Test of multiple comparisons with Dunn's correction (adjusted p-value)

In Table 2, a significant difference was observed between the lesion area in different treatment intervals. After the use of Dunn's test and the correction of the adjusted p-value, the main variation was found between the fifth and the twelfth week (adjusted p-value=0.032), which indicates that, at this moment in the treatment, a statistically significant reduction took place in the area of the lesions treated with 2% papain gel.

**Figure 1.**
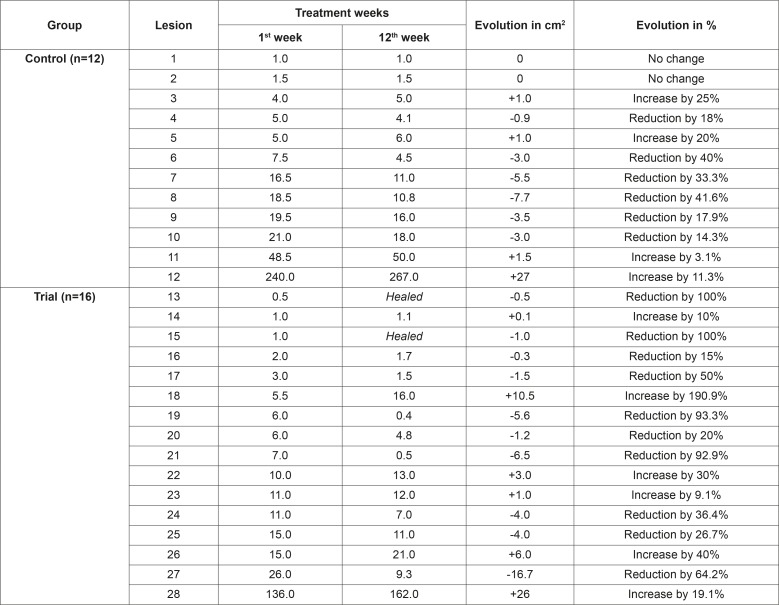
Evolution of venous ulcer area according to treatment group. Niterói, RJ, Brazil, 2014

Figure 1 displays the area of each lesion studied in the first and final week of the treatment.

Figure 1 illustrates that the area was reduced in 62.5% of the lesions in the Trial Group, with two wounds (12.5%) that healed completely. In 37.5% of the ulcer, the area increased at the end of the treatment. In the Control Group, the area was reduced in 50% of the lesions, increased in 33.3% and 16.7% maintained the same area that existed at the start of the treatment.

As regards the secondary outcomes, the slough tissue, unfavorable to healing, showed a statistically significant reduction between the first and the 12^th^ week of treatment, in the Trial Group (p-value=0.001) as well as in the Control Group (p-value=0.004). The granulation tissue, which can heal, showed a significant increase between the first and twelfth week of treatment in the Trial Group (p-value=0.021) and in the Control Group (p-value=0.031).

The epithelialized tissue, which represents the closing of the wound, showed a significant increase in the Trial Group only (p-value=0.004), while the Control Group showed a p-value of 0.063. It is highlighted that two ulcers (12.5%) in the Trial Group had healed completely at the end of the treatment.

As regards the amount of exudate, most ulcers (68.8%) in the Trial Group showed a small amount of exudate during the first visit to the clinic. During the last visit, 37.5% of the lesions showed a small amount; 37.5%, moderate; 12.5%, large and 12.5% absence of exudate, without significant difference at the end of the study (p-value=0.727). In the Control Group, 50% of the lesions showed a small amount of secretion at the start of the study; 25%, moderate and 25% large. At the end of the study, 58% of the lesions showed a small amount of exudate=0.750).

## Discussion

The analysis of the sociodemographic data showed the prevalence of retired people, as 22.2% of the sample were disability retirees, due to the chronic ulcers, which can cause the early distancing from work life, affect the quality of life and put a relevant strain on the country's health and social security systems.

The low level of education, associated with the participants' precarious socioeconomic condition, contributes to the reduced access to information on the prevention and care in venous ulcer treatment. It should be highlighted that all study participants suffered from chronic venous insufficiency: 61.1% suffered from systemic arterial hypertension and 11.1% from diabetes mellitus. These factors reveal the need for a high-quality public health service that offers treatment and monitoring to the patients, so that they turn into active agents of their health condition^17^.

In the assessment of chronic venous ulcers, two items serve as significant predictors of healing, the area and how long the lesion has existed. The area of the lesions in this study is in accordance with the Brazilian standard, with 89.3% of the ulcers measuring 26cm^2^ or less^18^^-^ 19. International studies consider wounds as large when measuring 10cm^2^ or more^15^. The Brazilian classification of wound areas is different though, with wounds measuring less than 50cm^2^ being considered small; between 50cm^2^ and 150cm^2^ medium; between 150cm^2^ and 250cm^2^ large, and extensive when measuring more than 250cm^2^ 20.

Other relevant information refers to how long the venous ulcers have existed. The majority (53.6%) started more than ten years earlier. It should be highlighted that, among the ulcers open for less than three years, all were relapsing wounds, which evidences the chronic nature of this type of lesion and the need to implement prevention measures in case of healing.

In a study developed in the USA that involved 165 venous ulcer patients and 12 weeks of monitoring, it was verified that, in cases of large and lengthy ulcers, rapid healing is impossible^21^. Another study evidenced that a good prognosis of venous ulcers depends directly on the size and evolution time of the ulcer. A wound measuring less than 10cm^2^ with an evolution time of less than 12 months has 71% chance of healing by the 24^th^ week of treatment, while wounds larger than 10cm^2^ with an evolution time of more than 12 months have a 22% chance of cure^22^.

The results demonstrated that the venous ulcers treated with 2% papain gel showed a significant reduction in the lesion area, especially between the 5^th^ and 12^th^ treatment week (adjusted p-value=0.032). Studies that examine the effectiveness of papain have been positive, but the majority does not contain a controlled comparison, or has involved the treatment of a wide range of wounds, instead of leg ulcer treatment^23^.

The Control Group that used the 2% carboxymethyl cellulose gel on the venous ulcers did not demonstrate a significant reduction in the lesion area (p-value=0.408) over the 12 weeks of treatment. In a review on the use of carboxymethyl cellulose dressings, evidence was found that suggests that these dressings are more effective in the healing of diabetic ulcers than other dressings, but this finding is doubtful due to the risk of bias in the original studies^24^.

The reduction in the amount of devitalized tissue, the secondary outcome, was achieved in the Trial Group (p-value=0.001) as well as in the Control Group (p-value=0.004). The devitalized tissue extends the inflammation phase and favors the proliferation of microorganisms and biofilms, retarding the entire healing process. Removing the devitalized tissue is recommended in wound treatment, preparing the wound bed before the dressing is applied. Papain is a chemical debriding agent due to its enzymatic action, which provokes the dissociation of the protein molecules, dissolving the necrotic tissue. Carboxymethyl cellulose gel, then, acts as an autolytic debriding agent, in which the devitalized tissue is destroyed by the action of enzymes in the exudate that is in contact with the ulcer^25^.

As regards the healthy tissue, the research data were favorable, with an increase in the amount of granulation tissue in the Trial Group (p-value=0.021) as well as in the Control Group (p-value=0.031). The increase in the granulation tissue was also observed in an international study of wounds treated using a product that associates papain and urea in comparison to wounds treated with collagenase^23^.

Only the Trial Group showed a significant increase (p-value=0.004) in the amount of epithelialized tissue during the treatment. Therefore, 2% papain gel showed a superior result to 2% CMC gel in this healing phase. The epithelialized tissue leads to the contraction and, consequently, the closure of the wound. An increase in this tissue indicates that the treatment used is favoring the tissue repair process^25^.

The amount of exudate in the two treatment groups varied between little and moderate in most lesions, without any significant change between the first and last treatment week. The lesser the amount of exudate, the better the healing conditions are, in view of the PUSH score^13^. An excessive quantity of exudate is associated with the critical colonization of the wound surface, with a systemic infection or persistent inflammation of the wound^25^. In a Brazilian study, most patients (60.0%) showed a moderate to large quantity of exudate^19^, differing from the result found in this research.

## Conclusion

After assessing the effectiveness of 2% papain gel compared to 2% carboxymethyl cellulose gel to treat chronic venous ulcers, it is concluded that the 2% papain gel was more effective concerning the primary outcome of this research, with a significant reduction in the lesion area in that group, particularly between the fifth and twelfth week of treatment.

As regards the secondary outcome, the reduction of exudate, no significant change was found between the groups, as the patients presented a little to moderate amount of exudate between the onset and end of the treatment, which is a positive characteristic of the group of patients attended at the Wound Clinic.

As regards the outcome reduction of devitalized tissue in the ulcer bed, the result was significant in the two groups. It was observed that both the Papain and the Carboxymethyl cellulose Groups showed favorable healing, with a reduction in the amount of devitalized tissue and the growth of granulation tissue.

One of the research limitations was the presence of outliers, with wounds larger than the pattern of the other wounds. Large and lengthy wounds are difficult to heal. These characteristics are appointed as aggravating factors for the treatment. Information with characteristics underlying the data can play a determinant role in the knowledge of the population the study sample belongs to and can guide specific clinical interventions.

In view of the importance of the theme, this study can contribute to the development of research protocols for wound assessment. Multicenter studies are needed to deepen the investigation of variables related to the effectiveness of papain gel.
